# The staging evolution dilemma: TNM-9 sharpens anatomical precision, but is biology still offstage?

**DOI:** 10.36416/1806-3756/e20250236

**Published:** 2025-07-22

**Authors:** Gustavo Faibischew Prado, Fernanda Kaori Fujiki, João Marcelo Lopes Toscano de Brito

**Affiliations:** 1. Comitê Científico de Câncer de Pulmão, Sociedade Brasileira de Pneumologia e Tisiologia, Brasília (DF), Brasil.; 2. International Association for the Study of Lung Cancer (Education Committee).; 3. Rede D’Or, São Paulo (SP), Brasil.; 4. Instituto do Câncer do Estado de São Paulo, São Paulo (SP), Brasil.

The 9th edition of the TNM classification for lung cancer (TNM-9),^1^ effective as of 2025, represents a further step toward enhanced prognostic accuracy and anatomically guided stratification of thoracic malignancies. Developed from extensive international databases, its primary innovations include the subdivision of N2 disease into N2a (single-level ipsilateral mediastinal nodal involvement) and N2b (multiple levels), as well as the distinction between extrathoracic metastases confined to a single organ system (M1c1) versus those involving multiple systems (M1c2). While these updates represent conceptual advances and improve statistical performance metrics, their direct clinical impact remains a subject of debate.^2^ ^-^ ^4^ The N2 subclassification provides more granular prognostic stratification: patients with N2a disease generally exhibit better overall survival than those with N2b. However, this difference may not always reach statistical significance across all cohorts, and preoperative prediction remains a challenge.^2^ ^,^ ^5^

In the study by Ferreira et al. (2025), featured in this issue of the *Jornal Brasileiro de Pneumologia*, the authors retrospectively applied TNM-9 to a cohort of 914 lung cancer patients previously staged according to the 8th edition. The reclassification led to notable stage shifts: 34 patients were downstaged, primarily from IIIB to IIIA, while 8 were upstaged from IIIA to IIIB. When evaluating the new N2a/N2b subclassification using EBUS-obtained samples, no statistically significant differences in overall survival (OS) were found between the two subgroups. This finding reflects limitations previously reported in surgical series, which highlight the difficulty of preoperatively predicting the true extent of mediastinal nodal involvement.^6^

Although the discriminative gap between stages IIIA and IIIB has narrowed-reflected in increasingly similar 5-year survival rates-the reclassification has introduced greater intra-stage homogeneity in certain scenarios. For instance, cases downstaged to IIB (e.g., T1N2aM0) exhibited outcomes consistent with others in that stage. Conversely, patients upstaged to IIIB (e.g., T3N2bM0) demonstrated better-than-expected survival, highlighting the persistent heterogeneity despite these adjustments.^1^ It is important to acknowledge that such changes may complicate the interpretation of historical data and affect eligibility criteria for clinical trials, thereby introducing new methodological challenges.

A key innovation with demonstrated prognostic impact in the study by Ferreira et al. lies in the subclassification of M1c metastases: patients with multi-organ involvement (M1c2) had significantly worse OS compared to those with metastases confined to a single organ system (M1c1), even within stage IVB. This observation supports recent studies and underscores the potential need to establish a new IVC stage for this subgroup, an aspect not currently addressed in the existing staging framework.^4^ ^,^ ^6^

The therapeutic implications of topographic refinement must also be taken into account. Patients with N2a disease, and some with N2b, particularly those with limited tumor burden, may be eligible for surgery following neoadjuvant chemoimmunotherapy. In contrast, those with bulky mediastinal disease are more likely to benefit from definitive chemoradiation followed by systemic therapies.^7^ ^,^ ^8^ However, in the absence of high-quality invasive staging, such as EBUS with systematic multistation sampling, this distinction may not be feasible, particularly in resource-limited settings.

It is important to note that landmark trials, such as CheckMate 816, AEGEAN, KEYNOTE 671, CHECKMATE 77T, IMpower010, KEYNOTE 091, and PACIFIC, have demonstrated significant improvements in event-free and overall survival when immune checkpoint inhibitors are incorporated into the treatment of patients with resectable and unresectable stage II and III disease, as compared to historical results.^9^ ^-^ ^15^ These novel strategies have the potential to reshape the survival landscape for patients whose clinical stages often overlap. However, access to these therapies varies considerably across institutions and countries, with socioeconomic disparities potentially confounding the generation and comparison of real-world survival data. As a result, datasets used to validate and refine stage groupings may reflect not only biological and anatomical factors but also be influenced by ‘systemic’ sociodemographic inequities. This introduces an additional layer of complexity to the interpretation of stage-based survival curves and raises the question of whether, in the era of precision medicine, anatomical staging alone can continue to serve as the core element for prognosis and therapeutic decision-making.

Despite these advances, TNM-9 still does not incorporate molecular, biological, or functional biomarkers, which are becoming ever more important for risk stratification and therapeutic decision-making. Predictive somatic alterations such as EGFR mutations and ALK rearrangements not only correlate with distinct prognoses but also guide the use of targeted therapies across all disease stages.^16^ ^,^ ^17^ Novel emerging tools, such as circulating tumor DNA (ctDNA) and pathologic response following neoadjuvant chemoimmunotherapy provide additional layers of prognostic-and potentially predictive-information, especially in the perioperative setting.^18^ ^,^ ^19^ Moreover, molecular profiling may enhance the staging of multifocal lesions by distinguishing synchronous primary tumors from metastases with poor prognosis, thereby enabling more individualized treatment approaches.^19^

As the field advances toward a more integrated and biologically informed approach to oncology, TNM-9 appears to serve as a necessary, yet interim and transitional framework. While it surpasses its predecessor in anatomical granularity, it falls short of capturing the full spectrum of individualized risks and treatment opportunities.

Future staging systems should integrate topographic, molecular, and clinical-functional dimensions not only to improve survival prediction but also to guide increasingly personalized therapeutic decision-making.

Such evolution will also demand updates to medical education and residency curricula, as the growing complexity of staging and therapeutics requires not only clinical acumen but also genomic literacy.

*In an era where staging can no longer be a static anatomical snapshot but must instead evolve into a dynamic map of therapeutic possibilities, TNM-9 offers a sharper lens, but still a narrow one.*
